# The risk of perchlorate and iodine on the incidence of thyroid tumors and nodular goiter: a case-control study in southeastern China

**DOI:** 10.1186/s12940-021-00818-8

**Published:** 2022-01-04

**Authors:** Huirong Wang, Yousheng Jiang, Jiayi Song, Huiwen Liang, Yuan Liu, Jiewu Huang, Pengliang Yin, Dongting Wu, Hang Zhang, Xinjie Liu, Dongxian Zhou, Wei Wei, Lin Lei, Ji Peng, Jianqing Zhang

**Affiliations:** 1 Shenzhen Center for Disease Control and Prevention, No.8 Longyuan Road, Nanshan District, Shenzhen, 518055 China; 2 School of Public Health, Southern Medical University, No.1023 Shatai Road, Baiyun District, Guangzhou, 510515 China; 3 Shenzhen Eye Hospital, Shenzhen Key Ophthalmic Laboratory, the Second Affiliated Hospital of Jinan University, No.18 Zetian Road, Futian District, Shenzhen, 518040 China; 4 Shenzhen People’s Hospital, No.1017 Dongmen North Road, Luohu District, Shenzhen, 518020 China; 5 Peking University Shenzhen Hospital, No.1120 Lianhua Road, Futian District, Shenzhen, 518036 China; 6 Shenzhen Center for Chronic Disease Control, No.2021 Buxin Road, Luohu District, Shenzhen, 518020 China

**Keywords:** Perchlorate, Iodine, Thyroid cancer, Nodular goiter, Thyroid function

## Abstract

**Background:**

The incidence rates of thyroid tumors and nodular goiter show an upward trend worldwide. There are limited reports on the risk of perchlorate and iodine on thyroid tumors, but evidence from population studies is scarce, and their impact on thyroid function is still uncertain. Therefore, the objective of this study was to investigate the association of perchlorate and iodine with the risk of nodular goiter (NG), papillary thyroid microcarcinoma (PTMC), and papillary thyroid carcinoma (PTC) and to assess the correlation between perchlorate and iodine with thyroid function indicators.

**Methods:**

A case–control population consisting of 184 pairs of thyroid tumors and nodular goiter matched by gender and age (±2 years) was recruited in this study. Serum and urine samples were collected from each participant. Thyroid function indicators in serum were tested by automatic chemical immunofluorescence, and perchlorate and iodine levels in urine were determined by ultra-high performance liquid chromatography tandem-mass spectrometry and inductively coupled plasma-mass spectrometry, respectively. Conditional logistic regressions and multiple linear regressions were used to analyze the associations.

**Results:**

Urinary perchlorate concentration was significantly higher in total cases, NG and PTC than in the corresponding controls (*P* < 0.05). Perchlorate was positively associated with PTC (OR = 1.058, 95% CI: 1.009, 1.110) in a non-linear dose–response relationship, but there was no association between perchlorate and NG or PTMC. Iodine was not associated with the risk of thyroid tumors and NG and did not correlate with the thyroid function indicators. Furthermore, perchlorate showed a positive correlation with thyroid stimulating hormone (TSH) at iodine adequate levels (*P* < 0.05), and a negative correlation with free triiodothyronine (FT3) and a positive correlation with thyroglobulin antibody (TgAb) at iodine more than adequate or excess levels (*P* < 0.05).

**Conclusions:**

Perchlorate can increase the risk of PTC in a non-linear dose–response relationship and disturb the thyroid hormone homeostasis and thyroid autoantibody levels.

**Supplementary Information:**

The online version contains supplementary material available at 10.1186/s12940-021-00818-8.

## Abstract (article highlights)

- Patients with thyroid tumor have higher perchlorate exposure than control.
- Perchlorate can increase the risk of papillary thyroid carcinoma.
- Perchlorate can disturb the homeostasis of thyroid function.
- Perchlorate was positively correlated with thyroglobulin antibody.

**Supplementary Information:**

The online version contains supplementary material available at 10.1186/s12940-021-00818-8.

## Background

The incidence of thyroid tumor has been sharply growing around the world, identification and characterization the risk factors for thyroid cancer and nodules have become a scientific hot topic in recent years. Thyroid tumors are divided into benign thyroid adenoma and malignant thyroid cancer (TC), and thyroid nodules (TNs) are a common benign proliferative disease of the thyroid gland with the detection rate in the general population as high as 65% [1]. Studies have shown that about 10% of TNs have the potential to progress to TC [1]. TC is the most common malignant tumor of the endocrine system and includes papillary carcinoma, follicular carcinoma, undifferentiated carcinoma, and medullary carcinoma. According to the ‘GLOBOCAN 2020’ published by the International Association of Cancer Registries (IARC) and World Health Organization (WHO), there were 586,202 new cases of global TC in 2020, of which 59.7% were in Asia [2]. Moreover, the total incidence of TC in the United States increased at a rate of 3% per year from 1974 to 2013 [3], and the number of TC cases in Denmark tripled between 1980 and 2014 [4]. Similarly, the age-standardized incidence of TC in China increased from 3.21/100,000 to 9.61/100,000 at an average rate of 3% per year from 2005 to 2015 [5]. Furthermore, the incidence rate of TC in Shenzhen, a typical fast modern and urbanized city of China, increased at an average rate of 11.33% per year from 2001 to 2015; the world population standard incidence rate increased from 3.55/100,000 to 17.97/100,000, ranking TC from the tenth to the second most common malignant tumor [6]. Although the incidence of TC has increased worldwide, the mortality rate is on a stable or even decreasing trend with the improvement of diagnostic and treatment techniques and early intervention of the disease [7]. Studies have shown that thyroid disease management costs in the United States are expected to triple by up to $3.5 billion/year by 2030, thus TC brings a great health and economic burden to human society [7]. There is an urgent need to explore the risk factors and potential environmental chemicals related to thyroid tumors so that thyroid disease can be controlled and prevented around the world.

It has been suggested that multiple factors, including genetics, radiation, iodine, autoimmune diseases, and complex and diverse environmental factors, such as environmental chemicals, may be the risk factors for thyroid tumors [8]. It has been considered that the rapid increase in the incidence of TC is the result of high-resolution ultrasound diagnostic methods and overdiagnosis. However, the effects of iodine intake and environmental pollutants such as perchlorate on thyroid tumors and their interactions cannot be ignored, but the evidence for such effects remains scarce. Perchlorate is mainly used for the production of rocket fuel and fireworks as oxidants in explosives [9]; with its high water solubility, stability, and difficulty to degrade, it is widely present in water, soil, air, and crops [10]. Most people are exposed to perchlorate through the dietary route [11, 12]. Industry wastewater and domestic sewage are the main sources of widespread perchlorate pollution in China, and most of the water and food samples (such as rice, milk, purified water) collected in China contain perchlorate in varying degrees [13, 14]. Previous studies have found that people living in the fireworks production area and electronic waste recycling area have higher concentrations of perchlorate in body fluids [9, 15]. While iodine plays an important role in the functioning of the thyroid gland and the thyroid hormone secretion system, perchlorate is structurally similar to iodide and is a competitive iodide inhibitor that can reduce iodine utilization by the thyroid gland [16, 17]. It has been demonstrated that perchlorate may lead to thyroid dysfunction or thyroid diseases by mediating the active transport of I^−^ on plasma membrane glycoprotein, thereby preventing I^−^ from entering thyroid follicular cells and interfering with the key steps of thyroid hormone biosynthesis [17]. However, the results have been reported on the effects of perchlorate and iodine on thyroid hormone levels are inconsistent. The effects of perchlorate on human thyroid function appear to be related to exposure dose, physiological feedback regulation mechanism, the combined effects of multiple endocrine disruptors, and nutritional iodine status, and so on [18–20]. Furthermore, there are limited reports showing that perchlorate exposure may increase the risk of thyroid papillary carcinoma (PTC) [21]. However, the relationships between perchlorate, iodine, and different pathological types of human thyroid tumors have not yet been reported.

Therefore, exploring the effects of perchlorate and iodine on three different pathological types of thyroid tumors and on thyroid function is urgently needed. The purpose of the present study was to investigate the effect of perchlorate and iodine on the risk of nodular goiter (NG), papillary thyroid microcarcinoma (PTMC), and PTC and to investigate the correlation with thyroid function indicators, so as to identify the key risk factors for thyroid disease. The study will provide a scientific basis for the prevention and control of thyroid tumors in future.

## Methods

### Study population

This study recruited 184 patients with thyroid tumors and NG and 211 controls among total 395 participants, of which 184 case–control pairs were matched by gender and age (± 2 years). The patients with newly diagnosed thyroid tumors and nodular goiter, not undergoing radiotherapy and chemotherapy, from September 2018 to September 2019 were recruited at a specialist clinic from two comprehensive third class hospitals in Shenzhen. They included 72 patients with NG, 46 patients with PTMC and 66 patients with PTC. All the three pathological types categorized as total cases. These patients met the “NCCN Clinical Practice Guidelines in Oncology: Thyroid Carcinoma” diagnostic guidelines issued by the National Comprehensive Cancer Network (NCCN) [22]. All participants had lived in Shenzhen for more than 6 months and signed the informed consent form. They were invited to participate in the face-to-face questionnaire survey. Blood and urine samples were taken from the participants. The control population was tested for five thyroid hormone indicators, two antibodies, and B-ultrasound, for those with normal thyroid function and ultrasound were defined as the control group. Exclusion criteria for the cases and controls were as follows: severe heart, liver, and kidney diseases; previous history of thyroid disease; use of iodine, thyroid hormones, and other drugs; pregnancy or recent use of contraceptives or estrogen; terminal cancer; use of radiotherapy and chemotherapy. The present study was approved by the Ethics Committee of the Shenzhen Center for Disease Control and Prevention and the two recruiting hospitals ([2019]012A).

### Sample collection and detection methods

Fasting venous blood was collected from the participants, and serum was separated by centrifugation at 3000 rpm for 15 min within 2 h and stored in cryogenic vials. The morning fasting middle-segment urine was collected and transferred into centrifuge tubes for storage. All samples were stored at − 80 °C in an ultra-low temperature refrigerator until analysis.

Serum levels of thyroid function indicators, including thyroid stimulating hormone (TSH), triiodothyronine (TT3), thyroxine (TT4), free triiodothyronine (FT3), free thyroxine (FT4), thyroid peroxidase antibody (TPOAb), and thyroglobulin antibody (TgAb), were measured by an automatic chemiluminescence analyzer (Maglumi 2000, Shenzhen New Industries Biomedical Engineering Co., Ltd., China). The reference range appears below: TSH: 0.30-4.50 μIU/mL; TT3: 0.69-2.15 ng/mL; TT4: 52.00-127.00 ng/mL; FT3: 2.00-4.20 pg/mL; FT4: 8.90-17.20 ρg/mL; TgAb: 0.00-95.00 IU/mL; TPOAb: 0.00-30 IU/mL [22].

Perchlorate in urine was detected by high-performance liquid chromatography with tandem mass spectrometry (HPLC-MS/MS). Specifically, 570 μL of urine sample was added into a 2-mL centrifuge tube; 30 μL of 1 μg/mL perchlorate isotope internal standard was added, mixed, and passed through 0.2-μm filter membrane. Finally, 5 μL of the solution was injected into the Infinity Lab Poroshell 120 HILIC-Z column (2.1 × 100 mm × 2.7 μm; Agilent Technologies, CA, USA). The standard substance of perchlorate was purchased from Beijing North Weiye Institute of Metrology and Technology (Beijing, China), and the internal standard substance was purchased from Shanghai ZZBIO Co. Ltd. (Shanghai, China). Two quality control samples were detected for every 20 samples, and the spiked recovery rate for samples was verified according to the low, medium, and high concentration of the samples. The limits of detection and quantification were 0.083 and 0.275 μg/L, respectively, and the spiked standard recovery was found between 93.2 and 115.5%.

The level of iodine in urine was evaluated by inductively coupled plasma mass spectrometry (ICP-MS, Agilent 7700 x, Agilent Technologies, USA). First, 1 mL of urine sample was accurately added into a 15-mL centrifuge tube, diluted to 10.0 mL with 0.25% tetramethylammonium hydroxide solution, oscillated on the vortex mixer, and then injected onto the ICP-MS. The standard substance for the analysis of iodine composition in freeze-dried human urine (purchased from the National Reference Laboratory for iodine deficiency disorders, Beijing, China) was used as the quality control sample to control the whole process of detection.

The levels of perchlorate and iodine in urine were corrected by urinary specific gravity (USG), and the formula is as follows [23] (C_*c*_: corrected concentration; C_*d*_: actual detected concentration; USG_*m*_: the median of USG in urine of case or control group; USG_*d*_: actual detected USG in individual urine):

$${C}_c={C}_d\times \frac{USG_m-1}{U{SG}_d-1}$$

### Statistical analyses

SPSS Statistics 25.0 (IBM, Chicago, USA) and R 3.6.3 (Lucent Technologies, USA) were used for statistical analysis of the data. Categorical variables were compared by the chi-square test. Mean ± SD (standard deviation) or median (interquartile range) was used to describe continuous variables. The Wilcoxon signed-rank test was used for intergroup comparisons, and the Kruskal–Wallis test was used for comparison of multiple groups.

Conditional logistic regression was used to assess the risk of thyroid tumors and NG related to urinary target variables (perchlorate, iodine) by using crude and adjusted odds ratios (ORs) and their corresponding 95% confidence intervals (CIs). According to WHO standards, iodine nutrition levels were divided into insufficient (< 100 μg/L), adequate (100–200 μg/L), more than adequate (200–300 μg/L), and excess (≥300 μg/L). Multiple conditional logistic regression analyses with *P* ≤ 0.10 as the inclusion criterion were conducted, and body mass index (BMI), smoking, and drinking were included as confounding variables. Restricted cubic spline regression with three knots (10th, 50th, and 90th percentiles) was used to evaluate the dose–response relationship between perchlorate exposure and the risk of thyroid tumors and NG. The relationship between perchlorate, iodine, and thyroid function indicators was analyzed only in the controls. Spearman’s rank correlation and multiple linear regression analysis were used to evaluate the relationship between urine target variables (perchlorate and iodine) and TSH, TT3, TT4, FT3, FT4, TPOAb, and TgAb in the control group. The variables with non-normal distribution (perchlorate, iodine, TSH, TPOAb, and TgAb) were natural log-transformed. Multiple linear regression model adjusted for gender, age, BMI, smoking, and drinking was established. All statistical significance level was set at *P* < 0.05 (two-sided).

## Results

Population characteristics are presented in Table 1. Among the 184 pairs of cases and controls, men and women accounted for 35.3 and 64.7%, respectively, with the female-to-male ratio of 1.8:1. The majority of patients were in the youth group (≤44 years old), followed by the middle-aged group (45–59 years old) and the elderly group (≥60 years old), accounting for 63.0, 26.6, and 10.4%, respectively. In the univariate analyses, the case group had a lower BMI and a higher proportion of individuals with drinking habit than the control group (*P* < 0.05).

**Table 1:** The characteristics of study population

Characteristics	Controls (*n* = 184)	Cases (*n* = 184)	*P*-value
Gender, n (%)			–
Male	65 (35.3)	65 (35.3)	
Female	119 (64.7)	119 (64.7)	
Age (years), n (%)			–
≤ 44	115 (62.5)	116 (63.0)	
45-59	51 (27.7)	49 (26.6)	
≥ 60	18 (9.8)	19 (10.4)	
BMI (kg/m^2^, Mean ± SD)	23.37 ± 3.45	22.23 ± 2.74	< 0.001
Educational level, n (%)			0.746
Elementary school and below	18 (9.8)	23 (12.5)	
Junior/ high school	88 (47.8)	80 (43.5)	
College Degree and above	78 (42.4)	81 (44.0)	
Duration of residence (years), n (%)			0.219
≤ 5	19 (10.3)	22 (12.0)	
5-10	53 (28.8)	38 (20.7)	
> 10	112 (60.9)	124 (67.4)	
Seafood, n (%)			0.325
< 1 times/week	27 (14.7)	31 (16.8)	
1-2 times/week	139 (75.5)	144 (78.3)	
> 2 times/week	18 (9.8)	9 (4.9)	
Smoking, n (%)	27 (14.7)	37 (20.1)	0.134
Drinking, n (%)	69 (37.5)	97 (52.7)	0.002

Note: *BMI* Body mass index, *SD* Standard deviation

*P*-value were derived from chi-square tests for categorical variables, Wilcoxon signed-rank test for continuous variables

The concentration distribution of perchlorate and iodine in urine is shown in Table 2. Perchlorate concentrations were higher in total cases compared to total controls (*P* < 0.05). After pathological classification, perchlorate concentrations were higher in NG and PTC than in the corresponding controls (*P* < 0.05). There was no significant difference among the three pathological types (*P* > 0.05). Furthermore, the levels of iodine in urine were not significantly different between total cases, NG, PTMC, PTC, and their corresponding controls (*P* > 0.05).

**Table 2:** Differences of perchlorate, iodine levels in urine between control and case group

Variables	Pathological type	Overall participants		Controls		Cases		*P*-value ^a^	*P-*value ^b^
		Median	25th-75th percentiles	Median	25th-75th percentiles	Median	25th-75th percentiles		
**Perchlorate (μg/L)**		11.27	6.89-18.49						
	Total cases			9.29	6.35-14.74	14.31	8.25-20.58	< 0.001	–
	NG			9.68	6.48-15.22	14.56	7.88-22.18	0.037	0.998
	PTMC			9.95	6.88-14.70	14.13	7.56-21.64	0.061	
	PTC			8.73	5.75-13.39	14.27	8.67-19.16	0.002	
**Iodine (μg/L)**		131.23	84.00-197.84						
	Total cases			131.59	82.13-183.25	131.23	84.46-214.92	0.153	–
	NG			122.67	66.63-171.41	130.19	79.60-205.02	0.165	0.836
	PTMC			138.58	100.43-181.96	148.34	95.15-213.52	0.589	
	PTC			130.35	84.74-189.80	126.97	88.82-215.71	0.697	

Note: Data are presented as medians and 25–75 percentiles. Perchlorate and iodine were corrected by urinary specific gravity (USG). *NG* nodular goiter, *PTMC* papillary thyroid micro carcinoma, *PTC* papillary thyroid carcinoma, *Total cases* including NG, PTMC and PTC

^a^ *P*-value were derived from Wilcoxon signed-rank test between the case and control group

^b^ *P*-value was derived from the Kruskal-Wallis test between the three types of pathological

Furthermore, potential confounding variables for thyroid tumors and nodular goiter have been evaluated (Supplementary Material, Table 1), and BMI, smoking, and drinking were included as confounding variables in multiple conditional logistic regression analyses (Table 3). The results showed that perchlorate was positively associated with the risk of total cases (OR = 1.041, 95% CI: 1.017, 1.066) after adjusting for covariates. The association between perchlorate and the different thyroid pathological types was further analyzed, and positive association with the risk of PTC was found (OR = 1.058, 95% CI: 1.009, 1.110), while no significant association was found between perchlorate and NG and between perchlorate and PTMC (*P* > 0.05). Moreover, there was no significant association between iodine and thyroid tumors and NG (*P* > 0.05).

**Table 3:** The association between perchlorate, iodine and thyroid tumors and nodular goiter by conditional logistic model

Variables	Total cases		NG		PTMC		PTC	
	ORs (%95CI)	*P*-value	ORs (%95CI)	*P*-value	ORs (%95CI)	*P*-value	ORs (%95CI)	*P*-value
N (case/control)	184/184		72/72		46/46		66/66	
Perchlorate	1.041 (1.017, 1.066)	0.001	1.034 (0.994, 1.074)	0.094	1.033 (0.996, 1.072)	0.084	1.058 (1.009, 1.110)	0.021
Iodine	1.000 (1.000, 1.000)	0.759	1.002 (0.998, 1.005)	0.303	1.000 (0.998, 1.001)	0.596	1.000 (0.999, 1.002)	0.518

Note: Perchlorate and iodine were corrected by urinary specific gravity (USG). Perchlorate, iodine, BMI, smoking, and drinking were included in the model. *OR* odds ratio, *CI* confidence interval, *NG* nodular goiter, *PTMC* papillary thyroid micro carcinoma, *PTC* papillary thyroid carcinoma, *Total cases* including NG, PTMC and PTC

**Fig. 1:**
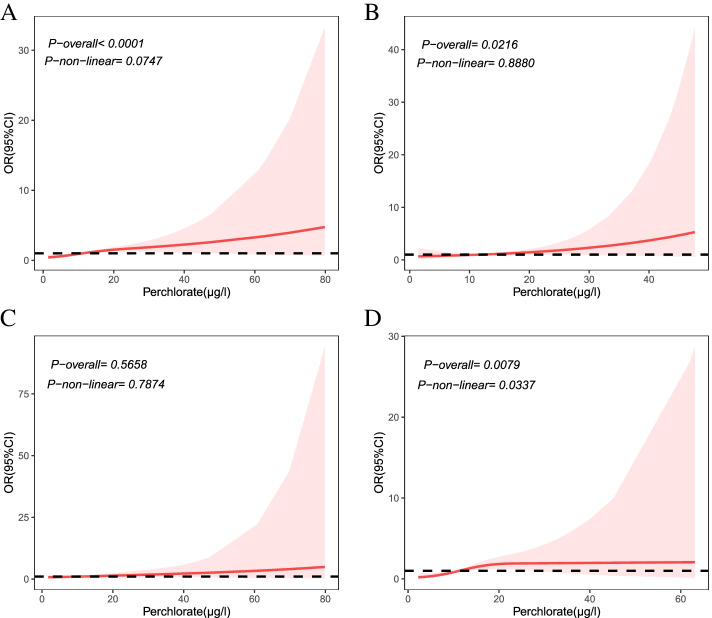
Dose-response relationship between perchlorate exposure and thyroid tumor and NG by restricted cubic spline (RCS). Perchlorate was corrected by urinary specific gravity (USG). **A** for total cases, **B** for NG, **C** for PTMC, **D** for PTC; estimates adjusted for BMI, smoking, drinking, and iodine; the red line represented the calculated ORs, the dotted line represented the baseline of OR = 1, the shades of red represent the 95% CI

Restricted cubic spline regression analysis showed a non-linear dose–response relationship between perchlorate and PTC (*P*-non-linear < 0.05) (Fig. 1 D and Supplementary Material, Table 2). The risk of PTC did not appear until perchlorate concentration reached 11.33 μg/L, and the ORs showed an increasing trend (1.008–1.924) for perchlorate concentrations between 11.33 and 27.04 μg/L. However, the risk trend for perchlorate on PTC was relatively flat and not monotonic, with the risk association disappearing when perchlorate concentrations was over 27.04 μg/L. The dose–response relationship between perchlorate and total cases and between perchlorate and NG was significant in the overall eq. (*P*-overall < 0.05) but also belonged to a nonmonotonic trend (Fig. 1 A and B). There was no statistically significant dose-response relationship between perchlorate and PTMC (Fig. 1 C).

Correlations between the thyroid function indicators and the levels of perchlorate and iodine were further analyzed in the control group. After adjusted for gender, age, BMI, smoking, and drinking, perchlorate correlated negatively with FT3 and positively with TgAb, with a β value of − 0.067 (95% CI: − 0.12, − 0.013) and 0.15 (95% CI: 0.0053, 0.30), respectively (Table 4). No significant effect of iodine on thyroid function indicators was observed (*P* > 0.05) (Table 4). Next, the effect of perchlorate on thyroid function indicators was further evaluated at three levels of iodine: insufficient, adequate, and more than adequate or excess (Table 5). The results showed that perchlorate positively correlated with TSH at iodine adequate levels, with a β value of 0.19 (95% CI: 0.040, 0.33). When the iodine level was at more than adequate or excess, perchlorate correlated negatively with FT3 and positively with TgAb, with a β value of − 0.12 (95% CI: − 0.23, − 0.0049) and 0.37 (95% CI: 0.023, 0.71), respectively.

**Table 4:** Multiple linear regression between perchlorate, iodine and thyroid function indicators in the control group

Indicators	Perchlorate		Iodine	
	β (%95CI)	*P*-value	β (%95CI)	*P*-value
TSH	0.045 (−0.043, 0.13)	0.322	−0.021 (− 0.067, 0.025)	0.370
TT3	0.0018 (− 0.037, 0.040)	0.929	−0.0083 (− 0.029, 0.012)	0.422
TT4	1.74 (− 0.82, 4.29)	0.185	− 0.37 (−1.71, 0.97)	0.588
FT3	−0.067 (− 0.12, − 0.013)	0.016	− 0.0043 (− 0.033, 0.024)	0.768
FT4	0.018 (− 0.0048, 0.040)	0.125	0.0067 (− 0.0051, 0.019)	0.268
TPOAb	0.014 (− 0.20, 0.23)	0.900	− 0.0077 (− 0.12, 0.11)	0.895
TgAb	0.15 (0.0053, 0.30)	0.044	−0.0075 (− 0.085, 0.071)	0.851

Note: Perchlorate and iodine were corrected by urinary specific gravity (USG); Perchlorate, iodine, gender, age, BMI, smoking, and drinking were included in the model. Perchlorate, iodine, TSH, TPOAb and TgAb were natural logarithm conversion. *CI* confidence interval, *TSH* thyroid stimulating hormone, *TT3* triiodothyronine; *TT4* thyroxine, *FT3* free triiodothyronine, *FT4* free thyroxine, *TPOAb* thyroid peroxidase antibody, *TgAb* thyroglobulin antibody

**Table 5:** Correlation between perchlorate and thyroid function under different iodine levels

Indicators	β	%95 CI for β	*P*-value
With inadequate iodine levels (*n* = 53)			
TSH	0.020	(−0.13, 0.17)	0.799
TT3	0.013	(−0.059, 0.086)	0.721
TT4	3.80	(−0.87, 8.47)	0.118
FT3	−0.041	(−0.14, 0.059)	0.429
FT4	0.020	(−0.022, 0.062)	0.358
TPOAb	0.41	(−0.037, 0.86)	0.079
TgAb	0.14	(−0.08, 0.35)	0.226
With adequate iodine levels (*n* = 87)			
TSH	0.19	(0.040, 0.33)	0.015
TT3	−0.013	(−0.082, 0.056)	0.715
TT4	2.46	(−2.00, 6.92)	0.283
FT3	−0.091	(− 0.19, 0.0044)	0.065
FT4	0.011	(−0.032, 0.055)	0.609
TPOAb	−0.18	(−0.55, 0.19)	0.349
TgAb	0.021	(−0.25, 0.29)	0.883
With more than adequate or excess iodine levels (*n* = 71)			
TSH	0.029	(−0.17, 0.23)	0.774
TT3	−0.071	(−0.14, 0.0021)	0.061
TT4	−2.55	(−7.88, 2.79)	0.353
FT3	−0.12	(−0.23, − 0.0049)	0.045
FT4	0.025	(−0.015, 0.066)	0.224
TPOAb	0.10	(−0.31, 0.51)	0.620
TgAb	0.37	(0.023, 0.71)	0.041

Note: Perchlorate and iodine were corrected by urinary specific gravity (USG); Perchlorate, iodine, gender, age, BMI, smoking, and drinking were included in the model. Perchlorate, iodine, TSH, TPOAb and TgAb were natural logarithm conversion. *CI* confidence interval, *TSH* thyroid stimulating hormone, *TT3* triiodothyronine; *TT4* thyroxine, *FT3* free triiodothyronine, *FT4* free thyroxine, *TPOAb* thyroid peroxidase antibody, *TgAb* thyroglobulin antibody

## Discussion

The present study demonstrated a positive association between perchlorate and the risk of PTC and perchlorate can disturb the homeostasis of thyroid function, but did not find an association between perchlorate and NG and PTMC. In addition, no association was found between iodine and the risk of thyroid tumors and nodular goiter, and thyroid function. The association of endocrine-disrupting compounds and iodide inhibitors with thyroid diseases has received worldwide attention in recent years. However, few population-based studies have examined the relationship between iodination inhibitors and thyroid diseases. To our knowledge, the present study is the first population-based study to simultaneously examine the association of perchlorate exposure with three thyroid neoplasma types (NG, PTMC, and PTC).

Our results showed a positive association between perchlorate exposure and the risk of PTC (OR = 1.058, 95% CI: 1.009, 1.110) with non-linear dose–response relationship (*P-*non-linear < 0.05). This is consistent with another small population study conducted in China, in which the association of perchlorate exposure and PTC was found (OR = 2.27, 95% CI: 1.03–5.03) [21]. The risk of perchlorate on PTC that we found was marginal and the effect of small sample size needs to be considered. In addition, humans are generally exposed to multiple endocrine disruptors in the external environment simultaneously [8], thus it is worth considering whether perchlorate act synergistically or antagonistically when combined with them on the thyroid. For example, Zhang et al., 2018 [21] included both perchlorate and thiocyanate and obtained a higher OR for the risk association than our study (2.27 > 1.058), so the combined effect of other thyroid disruptors on thyroid tumors cannot be excluded. It is worth noting that the initial concentration of perchlorate associated with the PTC risk was between the median value and the *P*_75_ level in the controls from the present study, which provided a statistical reference for the human exposure threshold. Animals studies have shown that exposure to perchlorate increases the size and number of thyroid follicles, which leads to structural changes in the thyroid gland [24]. However, previous studies have not determined whether perchlorate is involved in the initiating or the promoting mechanism in thyroid tumors. In the present study, we found a positive association between perchlorate and NG in univariate model, but this association disappeared in multifactorial model. It is possible that perchlorate is indeed not associated with NG, or the association may have been masked by some confounding factors not taken into account in our study. A report on perchlorate drinking water exposure test in rats from 2 weeks prior to cohabitation to lactation day 10 found that thyroid weight increased in female pups at 1.0 mg/kg/d ammonium perchlorate (AP) exposure compared to controls on day 22 of lactation [25]. Moreover, increased thyroid weight and decreased colloid in female rats and male/female pups were observed at 30.0 mg/kg/d AP exposure, further follicular hypertrophy was induced in female rats, suggesting that perchlorate may impair thyroid cells and alter thyroid morphology with gender sensitivity [25]. NG is also an irreversible pathological change that can be transformed from a diffuse goiter, thus the association of perchlorate with NG can be further investigated. In addition, no statistical association of perchlorate with the risk of PTMC was found either. Compared with PTC, PTMC is clinically asymptomatic, smaller in diameter (< 10 mm), and has a much lower mortality rate [26]. Given that we found that perchlorate exposure was associated with the risk of PTC but not PTMC, perchlorate exposure likely influences tumor size and probably acts as a promoter of TC rather than an initiator.

Furthermore, BMI was found to be a protective factor for thyroid tumors and NG, while drinking was a risk factor in this study. Similar findings were reported in Mijović T et al., 2009 [27] and 2010 to 2011 Korean National Health and Nutrition Examination Survey (KNHANES) [28]. However, recent studies show that BMI is positively correlated with papillary and follicular carcinomas of the thyroid, and this may be related to elevate TSH induced by obesity, chronic inflammatory response and oxidative stress, of which elevated TSH is considered a risk factor for TC [29]. Moreover, the study by Yeo et al. showed that smoking and drinking were negatively correlate with TC, which may be related to the lowering of TSH induced by smoking and drinking, as well as the anti-estrogenic effects [30]. These above findings suggest that confounding factors such as BMI, smoking, and drinking may affect the development of thyroid tumors and have been controlled in the following multivariate analysis of this study.

The effects of perchlorate on the indicators of thyroid function were assessed in this study, and the results showed that perchlorate was negatively correlated with FT3 and positively correlated with TgAb, and only positively correlated with TSH when iodine at adequate levels. According to the National Health and Nutrition Examination Survey (NHANES) in the United States, perchlorate was positively associated with TSH only in women aged above 12 years, regardless of whether iodine was adequate or not; in contrast, perchlorate was negatively associated with TT4 only in iodine deficiency status, suggesting an association between perchlorate and hypothyroidism [18]. However, perchlorate has been found to be positively associated with TSH in pregnant women only when co-exposed with thiocyanate and nitrate rather than independently [31]. A subchronic toxicity test study in rats showed that AP exposure was negatively associated with TT3 and TT4 and positively associated with TSH, which occurred only at doses of up to 10 mg/kg/day of AP exposure [32]. However, no significant changes in thyroid function were found in both trials from 9 healthy male volunteers for 2-week exposure at perchlorate dose 10 mg/d and 13 healthy volunteers for 6-month at exposure levels of 3 mg/d and 0.5 mg/d, and the small sample size for the above two studies may influence the statistical power. Nevertheless, effects of high-dose perchlorate exposure or lower doses of more prolonged perchlorate exposure on thyroid function haven’t been demonstrated in the general population till now [33, 34]. This indicates that perchlorate interference with TSH is also influenced by gender, exposure dose, and combined effects with other NIS inhibitors. We found the negative correlation of perchlorate and FT3 but not the positive correlation between perchlorate and TSH at the same iodine level, which may be related to sample size limitation or other confounding factors. In fact, it has been reported that TSH can stimulate the transcription and biosynthesis of NIS [17]. The above results might suggest the following possible mechanism: perchlorate competes with iodine for NIS to reduce iodine uptake in the thyroid gland, thereby inducing a decrease in FT3, which can reflect TT3 or TT4 decrease when not accompanied by thyroid hormone binding protein decrease, and increasing the secretion of TSH under the regulation of a negative feedback mechanism, while TSH restores the iodide level in thyroid cells by upregulation of NIS. In addition, the association of thyroid hormones and thyroid autoimmune antibodies with the risk of thyroid tumors has been reported [35, 36]. It has been shown that TSH can activate the phospholipase C/protein kinase C (PLC-PKC) signaling pathway to stimulate the secretion of vascular endothelial growth factor (VEGF) and induce neoangiogenesis, thereby promoting the proliferation of TC cells [37], which suggests that elevated TSH due to perchlorate exposure may have a role in promoting TC.

Both TPOAb and TgAb are markers of thyroid autoimmunity and can assist in the prediction of thyroid disease, such as hypothyroidism and prognosis of hyperthyroidism treatment [38]. Our study found a positive correlation between perchlorate and TgAb, suggesting that perchlorate may be associated with thyroid cell damage. However, since autoimmune thyroid disease usually requires a combination of positive TgAb and TPOAb for diagnosis, the effect of perchlorate on thyroid autoimmunity needs to be further verified. In particular, TgAb can also be used as a surveillance indicator for differentiated thyroid cancer (TDC) and has important implications for predicting the prognosis of TDC.

For iodine, we found no association with the risk of thyroid tumors and NG. Wang et al., 2014 pointed out that high iodine levels significantly correlate with the occurrence of benign and malignant thyroid tumors and the aggressiveness of PTC [39]. However, the median urinary iodine concentration in their study was 331.33 and 466.23 μg/L for thyroid nodules and TC, among which the iodine excess accounted for 62.75 and 66.99%, respectively, and much higher than that in our study [39]. In the present study, the median urinary iodine concentration was 130.19, 148.34 and 126.97 μg/L for NG, PTMC and PTC, respectively, while only 12.50 and 7.14% of patients with NG and TC (including PTMC and PTC) were at iodine excess levels (Supplementary Material, Table 4). Thus, the result of correlation between iodine and thyroid tumor from high iodine levels at iodine excess was no comparability with the present study. Several previous studies have demonstrated that the relationship between iodine and thyroid tumors is more evident at ultra-low and ultra-high intakes. For instance, experimental research revealed that ultra-low (1.0 × 10^− 6^ mol/L) and ultra-high dose (1.0 × 10^− 3^ mol/L) of iodine induced high expression of the SPANXA1 gene and promoted TC cell proliferation and apoptosis [40]. Furthermore, meta-analyses of animal and human studies have shown that both chronic iodine insufficiency and excess were risk factors for TC, but iodine intake was not a significant independent factor in the development of thyroid disease [41]. This is consistent with two epidemiological studies suggesting that high iodine intake may play a role in the tumor size and capsular invasion compared with the occurrence of PTC [42, 43].

Iodine plays an important role in the synthesis of thyroid hormones. However, no correlation between iodine levels and the thyroid function indicators was found in this study. This is similar to a previous cross-sectional study in China, which found no significant association between iodine levels and TSH, FT3, or FT4 [44]. Similarly, a large population study of over 7000 participants (NHANES) found no correlation between iodine levels and TSH and TT4 [45]. In contrast, studies based on complete food frequency surveys to assess iodine levels among young people residing in the mountains of the western United States have shown that iodine levels positively correlate with TSH [46]. The great variation among the studies may reflect the physiological regulation of normal human thyroid, where thyroid hormones are not significantly affected unless the thyroid gland is overloaded [47]. In addition, Wang et al., 2019 showed that both iodine deficiency and iodine excess are risk factors for autoimmune thyroid diseases [48]. Although both TgAb and TPOAb levels in the present study increased with the increase in iodine nutrition level, their variance at different iodine nutrition levels was not statistically significant and the antibody levels were still within the normal range (Supplementary Material, Table 5). It has been suggested that iodine is more likely to induce thyroid autoimmunity through unmasking a cryptic epitope on thyroglobulin [49]. The results of above study were only obtained from a single measurement. In fact, as individual iodine levels are closely related to the diurnal variation of dietary iodine content [50], it is even more important to observe fluctuations in thyroid hormones in the population by monitoring iodine nutrition levels over time.

The source of perchlorate exposure for the recruited population was analyzed in this study. The median perchlorate concentration in this study (11.27 μg/L) was notably higher than that of 6044 participants of the general population included in the NHANES (3.9 μg/L) [19]; however, the US study included not only multiregional and multiethnic populations but also adolescent population, suggesting that demographic and geographical variations may contribute to the differences in perchlorate exposure. Human perchlorate exposure has been related to the living environment and dietary intake. Studies of people living in fireworks production sites have shown that the mean concentration of perchlorate in urine is 16 times higher than that in the present study [9]. Research has supported that food intake contributes 86% to perchlorate exposure [51]. Although there have been no reports of perchlorate contamination in Shenzhen foods, limited studies have shown that perchlorate residues were found in vegetables, fruits, grains, and dairy products, especially in leafy vegetables (spinach) [10]. Moreover, the study of perchlorate concentration in bottled water and groundwater from 15 cities in China showed that the highest concentration (54.4 μg/L) was detected in Hengyang, Hunan Province—a fireworks production site, while Shenzhen was at the lowest concentration (0.14 μg/L) [13], suggesting that drinking water may not be the primary source of perchlorate exposure of the recruited population. Thus, the main source of perchlorate exposure in Shenzhen residents was more likely to be food intake. Further investigation on perchlorate exposure for Shenzhen residents is needed in the future.

There are some strengths and limitations in this study. First, cases with three pathological types of thyroid tumor and corresponding controls were matched by gender and age and recruited based on strict inclusion and exclusion criteria. Second, the authenticity and accuracy of data were ensured under strict quality control measures. The confounding variables were adjusted for using a multifactorial model. This study was the first to compare the effects of perchlorate exposure on the three types of thyroid neoplasms. Thus, the findings provide important etiological evidence for thyroid tumor and nodular goiter in order to improve thyroid disease prevention in future. However, there are some limitations to this study. First, the sample size in different pathological types was small, especially in the PTMC group. Second, the levels of perchlorate and iodine in urine were based on a single measurement as it is difficult to reflect long-term human burden levels in a cross-sectional study. However, the present study found the association of perchlorate with PTC and first indicated that perchlorate has no statistical association with the risk of NG and PTMC. In future, population-based cohort studies with precisely monitored iodine intake through long-term follow-up in the exposed populations from perchlorate-contaminated areas should be conducted.

## Conclusions

We found that perchlorate can increase the risk of PTC in a non-linear dose–response relationship, but no significant associations with NG and PTMC. Moreover, perchlorate can disturb thyroid hormone homeostasis and thyroid autoantibody levels. However, the association between iodine and thyroid tumors, NG, and thyroid function indicators has not been found in this study. The present study provided important population evidence for the identification of the risk factors of thyroid tumors and NG from the exogenous environment chemical exposure.

## Supplementary Information

**Additional file 1: Table S1**. The influencing factors of thyroid tumor and nodular goiter in univariate logistic regression model. **Table S2**. Dose-response relationship between perchlorate exposure and thyroid tumors and nodular goiter based on restricted cubic spline (RCS). **Table S3**. Correlation analysis of perchlorate and iodine with thyroid function indicators. **Table S4**. Distribution of people at different iodine nutrition levels. **Table S5**. Thyroid autoimmune antibodies concentration in the control group at different iodine nutrient levels.

## Data Availability

The dataset generated during and/or analyzed during the current study are not publicly available due to ethics reason.

## Abbreviations

BMI: Body mass index
OR: Odds ratio
CI: Confidence interval
NG: Nodular goiter
PTMC: Papillary thyroid microcarcinoma
PTC: Papillary thyroid carcinoma
TC: Thyroid cancer
TNs: Thyroid nodules
TSH: Thyroid stimulating hormone
TT3: Triiodothyronine
TT4: Thyroxine
FT3: Free triiodothyronine
FT4: Free thyroxine
TPOAb: Thyroid peroxidase antibody
TgAb: Thyroglobulin antibody
USG: Urinary specific gravity
NIS: Sodium/Iodide Symporter
AP: Ammonium perchlorate
TDC: Differentiated thyroid cancer
NHANES: National Health and Nutrition Examination Survey
KNHANES: Korean National Health and Nutrition Examination Survey
