# Supplementary material for: The risk of perchlorate and iodine on the incidence of thyroid tumors and nodular goiter: a case-control study in southeastern China
Source: Environ Health. 2022 Jan 4;21:4. doi: 10.1186/s12940-021-00818-8 (PMC8725411; doi:10.1186/s12940-021-00818-8)
Supplement: Supplementary file 1 — Additional file 1: Table S1. The influencing factors of thyroid tumor and nodular goiter in univariate logistic regression model. Table S2. Dose-response relationship between perchlorate exposure and thyroid tumors and nodular goiter based on restricted cubic spline (RCS). Table S3. Correlation analysis of perchlorate and iodine with thyroid function indicators. Table S4. Distribution of people at different iodine nutrition levels. Table S5. Thyroid autoimmune antibodies concentration in the control group at different iodine nutrient levels. [file 12940_2021_818_MOESM1_ESM.docx]

**Supplementary Material Table 1.** The influencing factors of thyroid tumor and nodular goiter in univariate logistic regression model

| Characteristic | Crude ORs (95% CI) | *P*-value |
| --- | --- | --- |
| **BMI** | 0.871 (0.805, 0.942) | <0.001 |
| **Educational level** |  |  |
| Elementary school and below | Reference | -- |
| Junior/ high school | 0.686 (0.332, 1.414) | 0.307 |
| College Degree and above | 0.812 (0.384, 1.715) | 0.584 |
| **Duration residence (years)** |  |  |
| ≤ 5 | Reference | -- |
| 5-10 | 0.604 (0.285, 1.282) | 0.189 |
| ≥ 10 | 0.961 (0.495, 1.865) | 0.907 |
| **Seafood** |  |  |
| < 1 times/week | Reference | -- |
| 1-2 times/week | 0.886 (0.471, 1.668) | 0.707 |
| > 2 times/week | 0.453 (0.174, 1.176) | 0.104 |
| **Smoking** | 1.769 (0.896, 3.493) | 0.100 |
| **Drinking** | 2.077 (1.301, 3.316) | 0.002 |
| **Urinary perchlorate in different pathological types** | | |
| Total cases (184/184) | 1.045 (1.022, 1.069) | <0.001 |
| NG (72/72) | 1.044 (1.008, 1.082) | 0.017 |
| PTMC (46/46) | 1.031 (0.997, 1.067) | 0.076 |
| PTC (66/66) | 1.067 (1.017, 1.119) | 0.008 |
| **Urinary iodine in different pathological types** | | |
| Total cases (184/184) | 1.000 (1.000, 1.000) | 0.373 |
| NG (72/72) | 1.003 (1.000, 1.006) | 0.074 |
| PTMC (46/46) | 1.000 (0.999, 1.001) | 0.502 |
| PTC (66/66) | 1.001 (0.999, 1.002) | 0.373 |

Note: Perchlorate and iodine were corrected by urinary specific gravity (USG).

Abbreviations: *OR* odds ratio, *CI* confidence interval, *BMI* body mass index, *NG* nodular goiter, *PTMC* papillary thyroid micro carcinoma, *PTC* papillary thyroid carcinoma

**Supplementary Material Table 2.** Dose-response relationship between perchlorate exposure and thyroid tumors and nodular goiter based on restricted cubic spline (RCS).

| Group | Percentile | Perchlorate (µg/L) | OR | 95% CI | |
| --- | --- | --- | --- | --- | --- |
|  |  |  |  | Lower | Upper |
| Total cases | 10^th^ | 4.96 | 0.580 | 0.384 | 0.876 |
|  | 50^th^ | 11.16 | 0.998 | 0.997 | 0.999 |
|  | 90^th^ | 26.37 | 1.745 | 1.261 | 2.413 |
|  | 92.5^th^ | 29.79 | 1.860 | 1.236 | 2.799 |
|  | 99^th^ | 60.96 | 3.332 | 0.863 | 12.868 |
| NG | 10^th^ | 4.88 | 0.768 | 0.356 | 1.653 |
|  | 50^th^ | 11.52 | 0.995 | 0.986 | 1.004 |
|  | 90^th^ | 25.96 | 1.909 | 0.991 | 3.674 |
|  | 92.5^th^ | 27.16 | 2.019 | 0.973 | 4.188 |
|  | 99^th^ | 47.04 | 5.133 | 0.642 | 41.072 |
| PTMC | 10^th^ | 5.09 | 0.81 | 0.477 | 1.371 |
|  | 50^th^ | 10.40 | 0.997 | 0.990 | 1.004 |
|  | 90^th^ | 33.87 | 1.949 | 0.856 | 4.437 |
|  | 92.5^th^ | 38.86 | 2.17 | 0.856 | 5.486 |
|  | 99^th^ | 69.85 | 4.01 | 0.367 | 43.742 |
| PTC | 10^th^ | 4.69 | 0.308 | 0.133 | 0.715 |
|  | 50^th^ | 11.33 | 1.008 | 1.003 | 1.014 |
|  | 90^th^ | 25.16 | 1.917 | 1.095 | 3.357 |
|  | 92.5^th^ | 27.04 | 1.924 | 1.013 | 3.655 |
|  | 99^th^ | 61.96 | 2.059 | 0.158 | 26.784 |

Note: Perchlorate was corrected by urinary specific gravity (USG); adjusted BMI, smoking, drinking and iodine.

Abbreviations: *OR* odds ratio, *CI* confidence interval, *NG* nodular goiter, *PTMC* papillary thyroid micro carcinoma, *PTC* papillary thyroid carcinoma, *Total cases* including NG, PTMC and PTC

**Supplementary Material Table 3.** Correlation analysis of perchlorate and iodine with thyroid function indicators.

| Indicators | Perchlorate | |  | Iodine | |
| --- | --- | --- | --- | --- | --- |
|  | r | *P*-value |  | r | *P*-value |
| TSH | 0.089 | 0.193 |  | -0.070 | 0.258 |
| TT3 | -0.030 | 0.648 |  | 0.008 | 0.907 |
| TT4 | 0.023 | 0.739 |  | -0.010 | 0.859 |
| FT3 | -0.15 | 0.024 |  | -0.010 | 0.869 |
| FT4 | 0.027 | 0.694 |  | 0.101 | 0.142 |
| TPOAb | 0.028 | 0.676 |  | 0.063 | 0.355 |
| TgAb | 0.081 | 0.239 |  | 0.047 | 0.491 |

Note: Perchlorate and iodine were corrected by urinary specific gravity (USG); Perchlorate, iodine, gender, age, BMI, smoking, and drinking were included in the model. Perchlorate, iodine, TSH, TPOAb and TgAb were natural logarithm conversion.

Abbreviations: *TSH* thyroid stimulating hormone, *TT3* triiodothyronine; *TT4* thyroxine, *FT3* free triiodothyronine, *FT4* free thyroxine, *TPOAb* thyroid peroxidase antibody, *TgAb* thyroglobulin antibody

**Supplementary Material Table 4.** Distribution of people at different iodine nutrition levels.

| Iodine nutrient levels | Overall participants | Controls | Cases | | |
| --- | --- | --- | --- | --- | --- |
|  |  |  | Total cases | NG | TC |
| Inadequate iodine | 124 (33.70) | 62 (33.70) | 62 (33.70) | 27 (37.50) | 35 (31.25) |
| Adequate iodine | 154 (41.85) | 84 (45.65) | 70 (38.04) | 25 (34.72) | 45 (40.18) |
| More than adequate iodine | 60 (16.30) | 25 (13.59) | 35 (19.02) | 11 (15.28) | 24 (21.43) |
| Excess iodine | 30 (8.15) | 13 (7.06) | 17 (9.24) | 9 (12.50) | 8 (7.14) |

Note: Note: Data presented by n (%). *NG* nodular goiter, *Total cases* including NG, PTMC and PTC, *TC* thyroid cancer, including PTMC and PTC

**Supplementary Material Table 5.** Thyroid autoimmune antibodies concentration in the control group at different iodine nutrient levels.

| Iodine nutrient levels | TPOAb | TgAb | *P*-value ^a^ | *P*-value ^b^ |
| --- | --- | --- | --- | --- |
| Inadequate iodine | 1.55 (0.80, 3.38) | 9.60 (7.60, 13.05) | 0.277 | 0.407 |
| Adequate iodine | 1.45 (0.38, 2.57) | 10.91 (7.43, 16.53) |  |  |
| More than adequate or excess iodine | 1.79 (0.78, 2.73) | 11.13 (6.96, 15.48) |  |  |

Note: Data presented by Median (Interquartile range)

^a^ *P*-value was derived from the Kruskal-Wallis test for TPOAb

^b^ *P*-value was derived from the Kruskal-Wallis test for TgAb

Abbreviations: *TPOAb* thyroid peroxidase antibody, *TgAb* thyroglobulin antibody
